# Examination

**Published:** 1871-09

**Authors:** 


### EXAMINATION.

One of the important points in the treatment of the teeth, is thorough examination. Is there not with a great many in the profession a very great deficiency in this respect? There may be some, perhaps many, who do not know what is meant by this expression. Does it mean a looking to ascertain if there is decay upon the teeth, that any one who should see the teeth would perceive? Does it mean this and nothing more? Would the physician be regarded as a good diagnostician, who would rest satisfied with the simple observation and knowledge of the fact, that his patient was pale, emaciated and weak, and go no further, and proceed at once to treat the patient, without even an effort to determine the nature of his disease, its depth and strength, its resources and hold upon the patient, and his ability to resist it?

No one of ordinary intelligence and common sense, would wish to place himself, or friend, or even his dog in the charge of such a physician; and yet, the great mass of the people are willing to place their teeth—priceless gems—for care and treatment in the hands of any one who may assume to call himself dentist, though he be as ignorant of what he attempts as the veryist fool.

Any one having a precious diamond or jewel, to be dressed and set, would be exceedingly careful in the selection of him who should do it. The stone would probably be sent hundreds or thousands of miles, to find the best skill for the work. It would amount to very little for the ignorant, inexperienced and unskilled, to procure a grindstone and put out his sign, "Samuel Slick, Lapidary." Samuel would probably starve to death sometime, before *any body*, however simple minded he might be, would venture to place a jewel in his hands for dressing and arranging.

Now every tooth is of more value by far than any ordinary diamond or jewel, and should receive more careful treatment.

These thoughts, and many others, have been suggested by two cases that have recently came under our observation. The one, a girl, fifteen years of age, some three or four weeks ago had her teeth examined by a man who has assumed for the last twenty-five years to be a dentist, and has been in the constant practice of the profession during that time. After making a thorough examination of the teeth, he informed the mother that the teeth were all free from decay and in good condition, but that they ought to be examined every year or two. Now, within ten days after this examination, the teeth came into our care, and there we found twenty-two well defined cavities of decay, nearly all of which were upon the proximal surfaces of the teeth, and but one of them broken down from the crown surface. In four of them the decay had penetrated the pulp chambers. In nearly all the decay was only indicated to superficial observation by a slight change of color immediately over the affected part. Several of the decays were through and beneath the fissures upon the masticating surfaces of the molars and bicuspids, the enamel not broken down in the fissures to any observable extent, and the color but slightly, if any changed. The mother was not satisfied with the first examination, and the latter was made.

Now, in this case, there was criminal ignorance or carelessness, perhaps both. Taking all the circumstances into account, the suspicious might attribute the conclusion of that examination to a motive of not the purest character.

The other case was a gentleman about twenty-five years of age, who had a number of teeth filled several years ago, called upon a professed dentist of many years practice, for an examination of his teeth, and especially of these fillings. After examination had, they were pronounced to be in good condition, the fillings preventing all decay so far as the filled cavities were concerned, at least. After a few days it was our lot to examine this case, and in seven or eight of the ten teeth that were filled, an instrument would readily pass down either through the gold or between the gold and the wall of the cavity, showing decay in active progress in each one, and especially at the entrance of the fissures. The pulp chambers of two of them were penetrated by the decay.

Now in both these cases, the mischief that would have resulted from their examination, would have been irreparable. The tendency in almost every such case would be to lead the patient into a false security, that would result in the loss of the teeth. If ignorance was the apology for the conclusions in these examinations, then there ought to be some criterion by which even the more obtuse could recognize such ignorance and avoid it. But if it be from carelessness, or an inward acquiescence that the teeth be lost, then such persons should be classed with and put upon a level with those reprobates who would do us a like amount of injury in any other respect. They ought to have the seal of condemnation stamped on their foreheads, that everybody might see and shun them. T.

